# Transcriptomic and bioinformatics analysis of the early time-course of the response to prostaglandin F2 alpha in the bovine corpus luteum^☆^^☆☆^

**DOI:** 10.1016/j.dib.2017.08.026

**Published:** 2017-09-01

**Authors:** Heather Talbott, Xiaoying Hou, Fang Qiu, Pan Zhang, Chittibabu Guda, Fang Yu, Robert A. Cushman, Jennifer R. Wood, Cheng Wang, Andrea S. Cupp, John S. Davis

**Affiliations:** a Olson Center for Women’s Health/Obstetrics and Gynecology Department, University of Nebraska Medical Center, 989450 Nebraska Medical Center, Omaha, NE 68198-9450, USA; b Biochemistry and Molecular Biology Department, University of Nebraska Medical Center, 985870 Nebraska Medical Center, Omaha, NE 68198-5870, USA; c Biostatistics Department, University of Nebraska Medical Center, 984375 Nebraska Medical Center, Omaha, NE 68198-4375, USA; d Bioinformatics and Systems Biology Core, University of Nebraska Medical Center, 985805 Nebraska Medical Center, Omaha, NE 68198-5805, USA; e Nutrition and Environmental Management Research Unit, Department of Agriculture, P.O. Box 166 (State Spur 18D)/USDA-ARS-PA-USMARC, Clay Center, NE 68933, USA; f Department of Animal Science, University of Nebraska—Lincoln, P.O. Box 830908, C203 ANSC, Lincoln, NE 68583-0908, USA; g Omaha Veterans Affairs Medical Center, 4101 Woolworth Ave, Omaha, NE 68105, USA

## Abstract

RNA expression analysis was performed on the corpus luteum tissue at five time points after prostaglandin F2 alpha treatment of midcycle cows using an Affymetrix Bovine Gene v1 Array. The normalized linear microarray data was uploaded to the NCBI GEO repository (GSE94069). Subsequent statistical analysis determined differentially expressed transcripts ± 1.5-fold change from saline control with *P* ≤ 0.05. Gene ontology of differentially expressed transcripts was annotated by DAVID and Panther. Physiological characteristics of the study animals are presented in a figure. Bioinformatic analysis by Ingenuity Pathway Analysis was curated, compiled, and presented in tables. A dataset comparison with similar microarray analyses was performed and bioinformatics analysis by Ingenuity Pathway Analysis, DAVID, Panther, and String of differentially expressed genes from each dataset as well as the differentially expressed genes common to all three datasets were curated, compiled, and presented in tables. Finally, a table comparing four bioinformatics tools’ predictions of functions associated with genes common to all three datasets is presented. These data have been further analyzed and interpreted in the companion article “Early transcriptome responses of the bovine mid-cycle corpus luteum to prostaglandin F2 alpha includes cytokine signaling” [1].

**Specifications Table**

**Table:** 

Subject area	*Biology*
More specific subject area	*Reproductive Biology*
Type of data	*Tables, graphs*
How data was acquired	*Collected empirical data, RNA microarray, Ingenuity Pathway Analysis, Panther Database*
Data format	*Raw data; Normalized, analyzed, and filtered data; curated bioinformatics predictions*
Experimental factors	*The estrous cycles of cows were synchronized using two injections of 25* *mg Lutalyse 11 days apart.*
Experimental features	*Post-pubertal multiparous female cattle (n = 16) of composite breeding were treated by intramuscular injection at midcycle (days 9–10) with saline (n = 4) or PGF2α (n = 12) (25* *mg Lutalyse). RNA was isolated from the corpus luteum and analyzed by microarray. Differentially expressed transcripts were subjected to bioinformatics pathway analysis.*
Data source location	*Lincoln, NE, USA; Omaha, NE, USA*
Data accessibility	*Raw data is in the public NCBI repository GEO (*GSE94069)*, curated bioinformatics predictions are presented within the article as tables*

## Value of the data

• This study provides the first transcriptomics analysis of the early time-course (0.5–4 h) of the response to prostaglandin F2 α (PGF2α) and extends previous observations on the global effects of PGF2α action in the bovine corpus luteum at 3 h and longer [2], [3].
• Prediction of upstream regulators and regulation of canonical pathways based on the transcriptome changes during the PGF2α short time-course.
• A complete list of differentially expressed transcripts grouped into self-organizing maps representative of signaling waves after PGF2α treatment.
• Canonical pathways and upstream regulators predicted by Ingenuity Pathway Analysis for genes common to three similar datasets [1], [2], [3].

## 1. Data

**Fig. 1:**
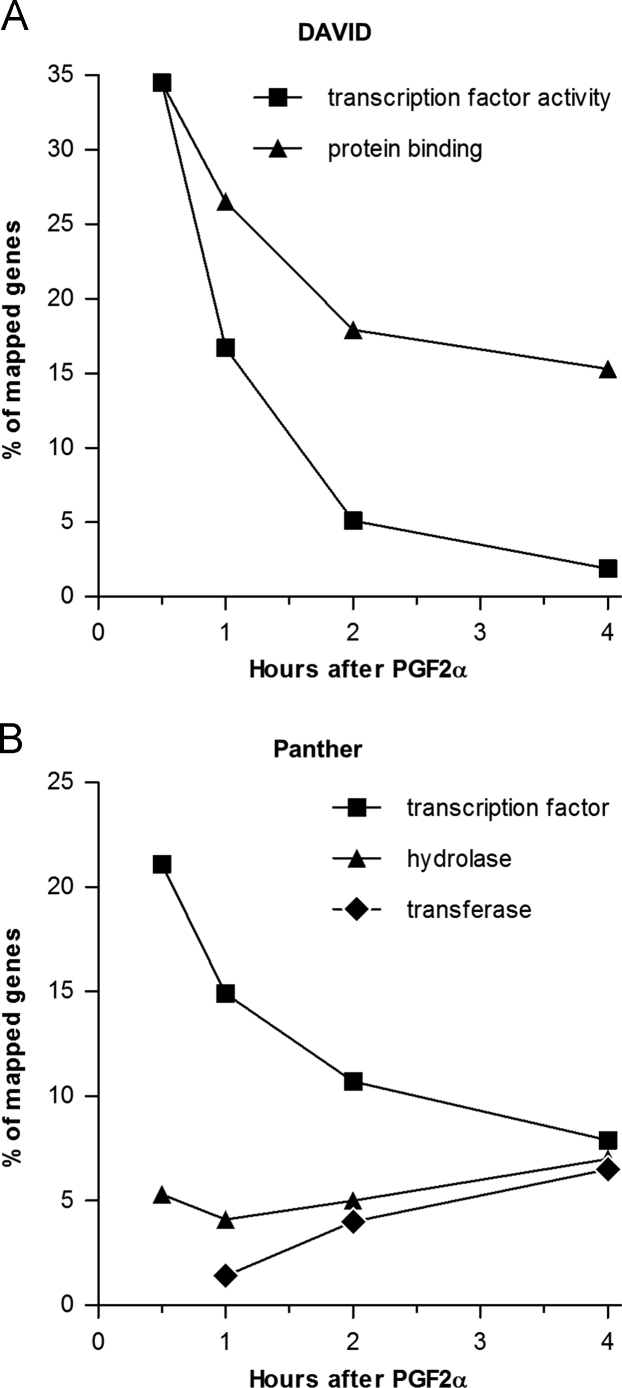
Biological process annotation of differentially expressed genes from each time point. **(A)** Percent of mapped genes with “transcription factor activity, RNA polymerase II core promoter proximal region sequence-specific binding” or “protein binding” annotations based on DAVID molecular function analysis (GOTERM_MF_ALL) of all differentially expressed genes from each time point. **(B)** Percent of mapped genes with “transcription factor (PC00218)”, “hydrolase (PC00121)”, or “transferase (PC00220)” annotations based on Panther Protein Class analysis of differentially expressed genes from each time point.
• Fig. 2 – Empirical characteristics of the female cattle used in the study

• The .cel and .chp files and normalized linear microarray data are available at the NCBI GEO repository: GSE94069
• Fig. 1 – Functional categorization of differentially expressed transcripts throughout the PGF2α time-course

**Fig. 2:**
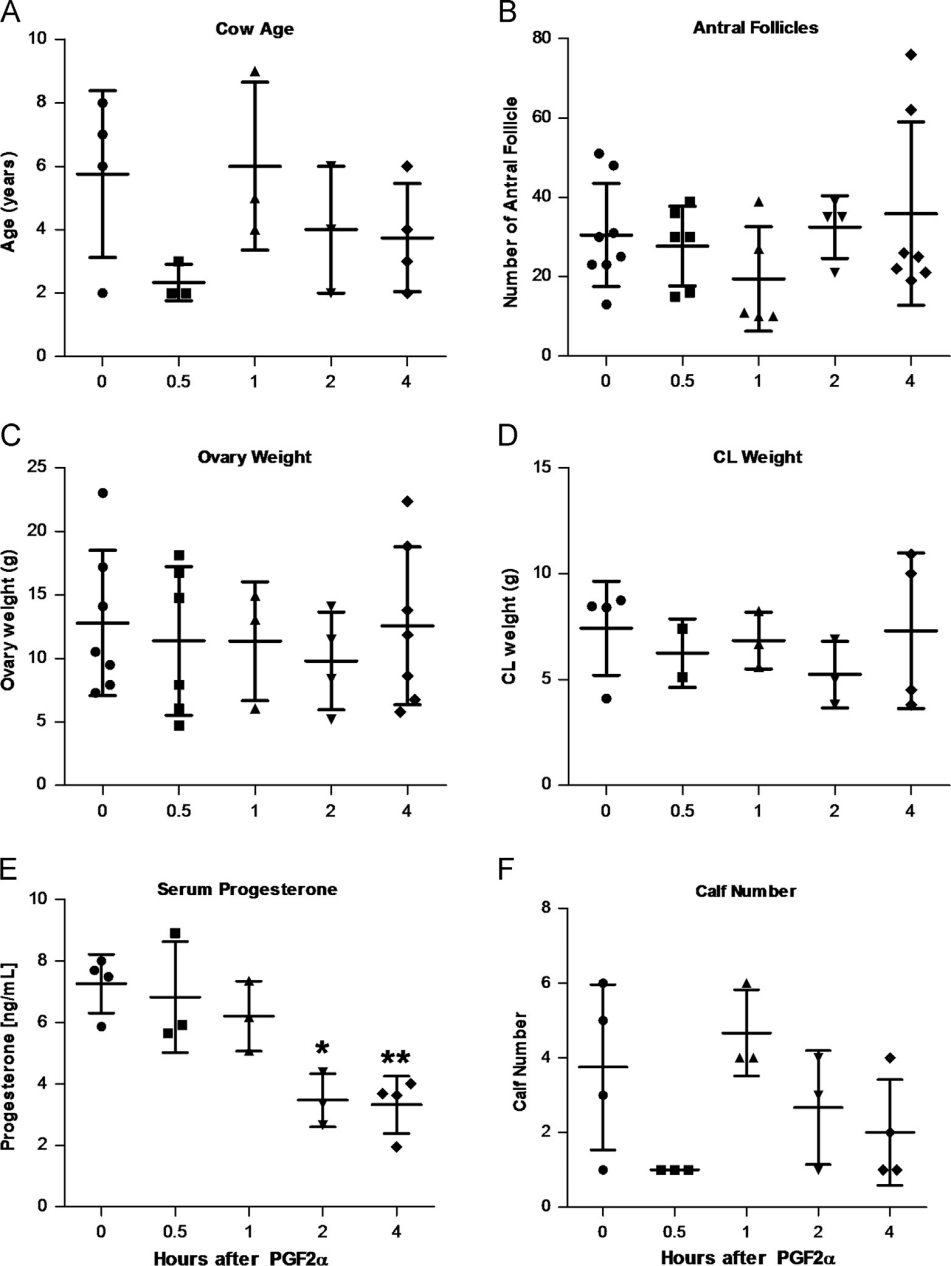
Physiological characteristics of the study animals. Mid-cycle cows were treated with 25 mg PGF2α for 0.5, 1, 2, and 4 h (n = 3/time point) or saline (n = 4). Symbols indicate individuals or each ovary, with mean±SD overlaid. **(A)** Age (in years) of cows at ovariectomy. **(B)** Number of antral follicles present on each ovary from study animals. **(C)** Total weight of each ovary from study animals. **(D)** Weight of corpus luteum (CL) from each study animal. **(E)** Previous number of calves from each study animal. **(F)** Serum progesterone concentrations of cows 0.5–4 h post-PGF2α treatment * *P* ≤ 0.05, ** *P* ≤ 0.01 compared to saline-treated animals using one-way ANOVA followed by Bonferroni's multiple comparison test.
• Table 1 – Ingenuity Pathway Analysis predicted canonical pathways involved during the PGF2α time-course

**Table 1:** Canonical pathways of PGF2α time course ⁎.

	**0.5** **h**		**1** **h**		**2** **h**		**4** **h**		
**Ingenuity Canonical Pathways**	**z-score**	***P-value (B-H)***	**z-score**	***P-value (B-H)***	**z-score**	***P-value (B-H)***	**z-score**	***P-value (B-H)***	**|Avg.| z-score**
Death Receptor Signaling							−2.71	3.23E-01	0.90
Integrin Signaling							−2.68	4.69E-01	0.89
UVA-Induced MAPK Signaling		2.51E-02		2.65E-01		7.96E-01	−2.50	1.64E-01	0.83
MIF Regulation of Innate Immunity		1.58E-02		8.71E-02		6.44E-01	2.45	3.44E-01	0.82
Retinoic acid Mediated Apoptosis Signaling						4.81E-01	−2.45	1.78E-01	0.82
Melanocyte Development and Pigmentation Signaling							−2.32	1.64E-01	0.77
TREM1 Signaling						7.89E-01	2.31	1.10E-01	0.77
CREB Signaling in Neurons							−2.18	4.61E-01	0.73
Aldosterone Signaling in Epithelial Cells		4.37E-02		7.76E-02		4.89E-01	−2.14	9.55E-02	0.71
NGF Signaling						8.39E-01	−2.13	4.79E-02	0.71
Calcium Signaling							−2.11	2.64E-01	0.53
Toll-like Receptor Signaling		2.24E-02		6.61E-02	2.00	2.99E-01	2.14	2.69E-02	1.03
ILK Signaling		5.25E-02	2.45	2.34E-02	1.63	7.31E-01		1.60E-01	1.36
Inflammasome pathway						7.55E-01	2.00	2.98E-01	0.50
MIF-mediated Glucocorticoid Regulation							2.00	5.16E-01	0.50
JAK/Stat Signaling		2.24E-02		2.45E-02		3.49E-01	−2.00	8.32E-02	0.50
Granzyme B Signaling						7.43E-01	−2.00	2.30E-01	0.50
Dopamine-DARPP32 Feedback in cAMP Signaling				6.15E-01			−2.00	4.50E-01	0.50
Signaling by Rho Family GTPases		7.59E-02	2.00	1.61E-01					0.67
LPS/IL-1 Mediated Inhibition of RXR Function		2.57E-01			2.00	7.43E-01	1.90	5.79E-01	0.97
LXR/RXR Activation					−1.34	4.99E-01	−2.32	1.61E-01	0.92
Cholecystokinin/Gastrin-mediated Signaling		2.45E-02	2.00	3.89E-02	2.65	2.99E-01	0.69	6.76E-02	1.33
TGF-β Signaling		2.24E-02		7.76E-02	2.00	5.45E-01	1.16	1.66E-01	0.79
Acute Phase Response Signaling		4.37E-02	1.00	7.76E-02	2.12	4.81E-01	1.53	1.23E-01	1.16
HMGB1 Signaling		3.09E-02	2.00	2.45E-02	1.89	3.44E-01	0.69	1.34E-01	1.14
Gαq Signaling				6.41E-01	−0.45	7.46E-01	−2.07	3.13E-01	0.63
Colorectal Cancer Metastasis Signaling		7.41E-02	2.00	1.61E-01	1.13	7.52E-01	−0.38	2.59E-01	0.69
Endothelin-1 Signaling		4.90E-02	2.00	8.71E-02			−0.66	3.94E-01	0.34
PI3K Signaling in B Lymphocytes		1.41E-02		1.22E-01	1.34	4.81E-01	−0.21	2.69E-02	0.28
Corticotropin Releasing Hormone Signaling		1.41E-02		3.89E-02		6.43E-01	−0.54	2.00E-01	0.18
IL-8 Signaling		5.62E-02	2.00	1.08E-01	0.45	8.39E-01	−1.09	4.00E-01	0.34
NRF2-mediated Oxidative Stress Response		1.41E-02	0.45	1.00E-02	0.38	1.75E-01	0.24	1.11E-01	0.27
Cardiac Hypertrophy Signaling		2.86E-01		2.88E-01	1.63	7.99E-01	−2.04	1.71E-01	0.10
IGF-1 Signaling		1.41E-02	1.00	1.91E-02	0.82	2.41E-01	−1.29	8.51E-02	0.13
IL-17A Signaling in Gastric Cells		1.41E-02		6.61E-02		4.99E-01		6.10E-01	

⁎ Original file contains pathways that contain at least one timepoint with | z-score| > 2. Pathways are sorted based on the |Avg| z-score from all four time points. |Avg| z-score is used solely for sorting of results, only z-scores for individual time points allow determination of pathway activation or inhibition. (B-H) Benjamini-Hockberg Multiple Testing Correction P-value limit set to 0.05
• Table 2 – Ingenuity Pathway Analysis predicted canonical pathways for the dataset comparison

**Table 2:** Canonical pathways of dataset comparison ⁎.

	**GSE94069**		**GSE23348**		**GSE27961**		
**Ingenuity Canonical Pathways**	**z-score**	***P-value (B-H)***	**z-score**	***P-value (B-H)***	**z-score**	***P-value (B-H)***	**|Avg.| z-score**
TREM1 Signaling	2.31	1.90E-01	4.24	3.55E-01	2.24	4.34E-01	2.93
p38 MAPK Signaling	1.34	7.94E-01	3.36	1.66E-01	2.53	1.75E-01	2.41
Acute Phase Response Signaling	1.53	1.19E-01	3.58	2.19E-01	2.12	6.24E-01	2.41
Dendritic Cell Maturation		1.13E-01	3.27	2.90E-01	1.41	5.18E-01	2.34
Inflammasome pathway	2.00	3.20E-01	2.65	5.13E-01		5.10E-01	2.33
MIF Regulation of Innate Immunity	2.45	3.52E-01	2.00	2.67E-01			2.23
CREB Signaling in Neurons	−2.18	4.67E-01					2.18
LPS/IL-1 Mediated Inhibition of RXR Function	1.90	5.85E-01	2.45	1.80E-01		2.26E-01	2.18
Role of IL-17F in Allergic Inflammatory Airway Diseases	1.27	4.68E-01	3.00	1.20E-01	2.24	1.35E-01	2.17
LXR/RXR Activation	−2.32	1.17E-01	−2.83	1.35E-01	−1.34	4.28E-01	2.16
Aldosterone Signaling in Epithelial Cells	−2.14	9.12E-01		1.75E-01		4.99E-01	2.14
Type I Diabetes Mellitus Signaling		6.11E-01	2.11	4.70E-01			2.11
IL-6 Signaling	1.23	2.57E-01	3.41	2.75E-01	1.67	3.27E-01	2.10
MIF-mediated Glucocorticoid Regulation	2.00	5.18E-01	2.00	1.61E-01			2.00
Granzyme B Signaling	−2.00	2.32E-01		6.98E-01		7.60E-01	2.00
Dopamine-DARPP32 Feedback in cAMP Signaling	−2.00	4.62E-01					2.00
Role of Wnt/GSK-3β Signaling in the Pathogenesis of Influenza					2.00	5.10E-01	2.00
Toll-like Receptor Signaling	2.14	2.57E-01	2.71	5.62E-01	1.00	6.24E-01	1.95
PI3K/AKT Signaling	2.13	2.57E-01	1.90	6.92E-01	1.67	3.83E-01	1.90
Actin Nucleation by ARP-WASP Complex			1.63	1.58E-01	2.00	5.10E-01	1.82
ILK Signaling		1.53E-01	2.32	5.25E-01	1.29	1.59E-01	1.81
Retinoic acid Mediated Apoptosis Signaling	−2.45	1.74E-01	-1.00	1.61E-01		7.26E-01	1.73
HMGB1 Signaling	0.45	1.90E-01	2.99	2.82E-01	1.67	3.45E-01	1.70
Regulation of Actin-based Motility by Rho			1.34	5.77E-01	2.00	7.60E-01	1.67
Rac Signaling		4.67E-01	2.14	1.70E-01	1.13	5.31E-01	1.64
Cholecystokinin/Gastrin-mediated Signaling	0.69	7.80E-01	2.31	2.57E-01	1.89	4.75E-01	1.63
VDR/RXR Activation	0.82	1.19E-01	1.67	2.34E-01	2.24	2.82E-01	1.58
NF-κB Signaling	0.54	2.57E-01	3.27	2.75E-01	0.91	4.34E-01	1.57
iNOS Signaling	1.00	1.14E-01	2.00	3.21E-01			1.50
Role of Pattern Recognition Receptors in Recognition of Bacteria and Viruses	−0.28	1.66E-01	3.21	4.37E-01		4.84E-01	1.47
Ephrin Receptor Signaling			0.82	3.93E-01	2.00	5.85E-01	1.41
Agrin Interactions at Neuromuscular Junction	0.38	5.27E-01	2.00	5.86E-01	1.63	1.59E-01	1.34
Tec Kinase Signaling	−1.21	5.31E-01	3.50	7.80E-01	1.41	6.59E-01	1.23
ERK5 Signaling	0.28	8.32E-01	1.41	5.75E-01	2.00	5.51E-01	1.23
Production of Nitric Oxide and Reactive Oxygen Species in Macrophages	−0.76	1.90E-01	3.15	3.39E-01			1.20
UVA-Induced MAPK Signaling	−2.67	1.19E-01		1.90E-01	0.45	5.51E-01	1.11
PI3K Signaling in B Lymphocytes	−0.21	2.57E-01	2.36	5.13E-01			1.08
Colorectal Cancer Metastasis Signaling	−0.56	2.18E-01	2.70	7.80E-01	1.00	3.70E-01	1.05
Basal Cell Carcinoma Signaling	−0.45	6.34E-01			2.45	3.70E-01	1.00
B Cell Receptor Signaling	−0.58	9.77E-01	2.83	8.71E-01	0.71	6.50E-01	0.99
Phospholipase C Signaling	−1.41	4.62E-01	3.32	5.32E-01	1.00	5.10E-01	0.97
Glioma Invasiveness Signaling	−0.30	2.69E-01	2.11	8.32E-01	1.00	1.38E-01	0.94
Oncostatin M Signaling	−0.45	4.23E-01	2.24	9.12E-01		4.95E-01	0.90
Neuregulin Signaling	−0.30	3.13E-01	2.00	5.81E-01		7.60E-01	0.85
JAK/Stat Signaling	−2.00	7.94E-01	0.33	8.32E-01			0.84
Calcium Signaling	−2.11	2.77E-01			0.45	1.38E-01	0.83
Role of RIG1-like Receptors in Antiviral Innate Immunity	−0.45	1.90E-01	2.00	5.75E-01			0.78
Type II Diabetes Mellitus Signaling	−0.58	3.36E-01	2.12	2.23E-01			0.77
PKCθ Signaling in T Lymphocytes	−1.41	1.66E-01	2.50	3.72E-01			0.55
NGF Signaling	−2.13	5.10E-01	2.33	2.85E-01	1.34	6.32E-01	0.51
Fcγ Receptor-mediated Phagocytosis in Macrophages and Monocytes	−1.73	3.97E-01	2.12	2.31E-01	1.13	4.28E-01	0.51
Role of NFAT in Regulation of the Immune Response	−1.50	3.75E-01	2.50	6.92E-01			0.50
Cardiac Hypertrophy Signaling	−2.40	1.69E-01	1.70	4.00E-01	2.14	4.34E-01	0.48
Death Receptor Signaling	−2.71	3.36E-01	0.28	3.89E-01	1.00	7.24E-01	0.48
Wnt/Ca+ pathway	−1.13	4.67E-01	0.45	2.92E-01	2.00	5.10E-01	0.44
Gαq Signaling	−2.36	4.40E-01	1.51	2.39E-01			0.43
CNTF Signaling	−2.11	1.33E-01	1.34	3.51E-01		5.26E-01	0.39
IL-8 Signaling	−1.90	4.19E-01	2.00	5.10E-01	0.58	4.70E-01	0.23
Integrin Signaling	−2.68	4.88E-01	1.51	4.99E-01	1.39	4.34E-01	0.07
Melanocyte Development and Pigmentation Signaling	−2.32	1.56E-01	1.34	4.90E-01	0.82	4.34E-01	0.05

⁎ Original file contains pathways that contain at least dataset with | z-score| > 2. Pathways are sorted based on the |Avg| z-score from all three datasets. |Avg| z-score is used solely for sorting of results, only z-scores for individual time points allow determination of pathway activation or inhibition. (B-H) Benjamini-Hockberg Multiple Testing Correction P-value limit set to 0.05
• Table 3 – Ingenuity Pathway Analysis predicted canonical pathways for the genes common to all datasets

**Table 3:** Canonical pathways of common genes ⁎.

**Ingenuity Canonical Pathways**	**z-score**	***P*****-value**	**Molecules**
Glioma Invasiveness Signaling	2.00	1.74E-03	*PIK3CA, ITGAV, PLAUR, CD44*
IL-6 Signaling	2.00	2.00E-03	*IL18, PIK3CA, SRF, CD14, IL33*
Acute Phase Response Signaling	2.00	2.57E-02	*IL18, PIK3CA, SERPINE1, IL33*
NF-κB Signaling	2.00	3.16E-02	*IL18, PIK3CA, BMP2, IL33*
PDGF Signaling	1.00	3.63E-03	*PIK3CA, SRF, SPHK1, PDGFC*
LXR/RXR Activation	−1.00	7.41E-03	*IL18, CD14, ARG2, IL33*
Atherosclerosis Signaling		1.05E-03	*IL18, TNFRSF12A, MMP1, IL33, PDGFC*
HIF1α Signaling		1.07E-03	*PIK3CA, LDHA, SLC2A1, MMP1, PDGFC*
GDP-glucose Biosynthesis		1.10E-03	*HK2, PGM5*
IL-10 Signaling		1.23E-03	*IL18, CD14, ARG2, IL33*
Hepatic Fibrosis / Hepatic Stellate Cell Activation		1.29E-03	*BAMBI, CD14, SERPINE1, AGTR1, MMP1, PDGFC*
Glucose and Glucose-1-phosphate Degradation		1.45E-03	*HK2, PGM5*
Bladder Cancer Signaling		2.34E-03	*CDKN1A, THBS1, MMP1, PDGFC*
Human Embryonic Stem Cell Pluripotency		2.57E-03	*INHBA, PIK3CA, SPHK1, BMP2, PDGFC*
TGF-β Signaling		3.24E-03	*INHBA, TGIF1, SERPINE1, BMP2*
Granulocyte Adhesion and Diapedesis		3.80E-03	*IL18, SDC4, CLDN1, MMP1, IL33*
Agranulocyte Adhesion and Diapedesis		4.79E-03	*IL18, SDC4, CLDN1, MMP1, IL33*
Role of Osteoblasts, Osteoclasts and Chondrocytes in Rheumatoid Arthritis		4.79E-03	*IL18, PIK3CA, BMP2, SPP1, MMP1, IL33*
Role of Tissue Factor in Cancer		1.07E-02	*PIK3CA, ITGAV, PLAUR, MMP1*
LPS/IL-1 Mediated Inhibition of RXR Function		1.12E-02	*IL18, CD14, HS3ST5, NR5A2, IL33*
VDR/RXR Activation		1.41E-02	*CD14, CDKN1A, SPP1*
Altered T Cell and B Cell Signaling in Rheumatoid Arthritis		1.41E-02	*IL18, SPP1, IL33*
Palmitate Biosynthesis I (Animals)		1.45E-02	*OXSM*
Fatty Acid Biosynthesis Initiation II		1.45E-02	*OXSM*
Toll-like Receptor Signaling		1.48E-02	*IL18, CD14, IL33*
Role of Hypercytokinemia/hyperchemokinemia in the Pathogenesis of Influenza		1.78E-02	*IL18, IL33*
Graft-versus-Host Disease Signaling		1.91E-02	*IL18, IL33*
Macropinocytosis Signaling		1.95E-02	*PIK3CA, CD14, PDGFC*
Hepatic Cholestasis		2.00E-02	*IL18, CD14, NR5A2, IL33*

⁎ Original file has pathways with P-value > 0.02 and sorted from largest to smallest based on z-score then smallest to largest P-value, Fisher's exact test P-value limit set to 0.05
• Table 4 – Comparison of bioinformatics tool predictions for the genes common to all datasets

**Table 4:** Comparison of bioinformatic tools ^⁎^.

	**DAVID (124/124)**	**IPA (116/124)**	**Panther (94/124)**	**String (93/124)**
**Canonical Pathways**	***P*****-value**	***P*****-value**	***P*****-value**	**False Discovery Rate**
TGF-beta signaling pathway	5.20E-03	3.24E-03	2.21E-02	2.94E-02
p53 signaling pathway	2.20E-02	3.89E-02		2.26E-02
Proteoglycans in cancer	1.50E-03			8.24E-03
HIF-1 signaling pathway	9.50E-03	1.07E-03		
ECM-receptor interaction	6.10E-03			8.24E-03
Bladder cancer	4.50E-02	2.34E-03		
Atherosclerosis Signaling		1.05E-03		
GDP-glucose Biosynthesis		1.10E-03		
IL-10 Signaling		1.23E-03		
Hepatic Fibrosis/Hepatic Stellate Cell Activation		1.29E-03		
Glucose and Glucose-1-phosphate Degradation		1.45E-03		
Glioma Invasiveness Signaling		1.74E-03		
Human Embryonic Stem Cell Pluripotency		2.57E-03		
PDGF Signaling		3.63E-03		
Granulocyte Adhesion and Diapedesis		3.80E-03		
Agranulocyte Adhesion and Diapedesis		4.79E-03		
Role of Osteoblasts, Osteoclasts and Chondrocytes in Rheumatoid Arthritis		4.79E-03		
Plasminogen activating cascade			7.05E-03	
LXR/RXR Activation		7.41E-03		
Role of Tissue Factor in Cancer		1.07E-02		
LPS/IL-1 Mediated Inhibition of RXR Function		1.12E-02		
VDR/RXR Activation		1.41E-02		
Altered T Cell and B Cell Signaling in Rheumatoid Arthritis		1.41E-02		
Palmitate Biosynthesis I (Animals)		1.45E-02		
Fatty Acid Biosynthesis Initiation II		1.45E-02		
Toll-like Receptor Signaling		1.48E-02		
Role of Hypercytokinemia/hyperchemokinemia in the Pathogenesis of Influenza		1.78E-02		
Graft-versus-Host Disease Signaling		1.91E-02		
Macropinocytosis Signaling		1.95E-02		
Hepatic Cholestasis		2.00E-02		
Coagulation System		2.40E-02		
LPS-stimulated MAPK Signaling		2.45E-02		
PPAR Signaling		2.45E-02		
Acute Phase Response Signaling		2.57E-02		
HER-2 Signaling in Breast Cancer		2.57E-02		
RNA degradation	2.60E-02			
Role of Cytokines in Mediating Communication between Immune Cells		2.69E-02		
Prostate Cancer Signaling		2.75E-02		
Aldosterone Signaling in Epithelial Cells		2.75E-02		
Trehalose Degradation II (Trehalase)		2.88E-02		
Pyruvate Fermentation to Lactate		2.88E-02		
Arginine Degradation I (Arginase Pathway)		2.88E-02		
NF-κB Signaling		3.16E-02		
tRNA Splicing		3.16E-02		
Cholecystokinin/Gastrin-mediated Signaling		3.31E-02		
Role of Oct4 in Mammalian Embryonic Stem Cell Pluripotency		3.80E-02		
Glucocorticoid Receptor Signaling		3.98E-02		
Nitric Oxide Signaling in the Cardiovascular System		3.98E-02		
Glioma Signaling		4.27E-02		
Urea Cycle		4.27E-02		
Arginine Degradation VI (Arginase 2 Pathway)		4.27E-02		
Pentose Phosphate Pathway (Non-oxidative Branch)		4.27E-02		
p38 MAPK Signaling		4.47E-02		
FXR/RXR Activation		4.68E-02
• Supplemental Table 1 – Ingenuity Pathway Analysis predicted upstream regulators involved during the PGF2α time-course
• Supplemental Table 2 – Ingenuity Pathway Analysis predicted upstream regulators for the SOMs
• Supplemental Table 3 – Ingenuity Pathway Analysis predicted diseases and functional annotations for the SOMs
• Supplemental Table 4 – Ingenuity Pathway Analysis predicted upstream regulators for the dataset comparison
• Supplemental Table 5 – Ingenuity Pathway Analysis predicted upstream regulators for the genes common to all datasets

## 2 Experimental design, materials and methods

### 2.1 Animals

Post-pubertal multiparous female cattle (n = 16) of composite breeding (½ Red Angus, Pinzgauer, Red Poll, Hereford and ½ Red Angus and Gelbvieh) were synchronized using two intramuscular injections of PGF2α (25 mg; Lutalyse®, Zoetis Inc., Kalamazoo Michigan, MI) 11 days apart. At mid-cycle (days 9–10), cows were treated with an intra-muscular injection of saline (n = 4) and subjected to a bilateral ovariectomy 0.5 h after the injection.

Cows were also treated with an intra-muscular injection of PGF2α (n = 12) and at each of four time points post-injection (0.5, 1, 2, and 4 h), three cows per time point were subjected to a bilateral ovariectomy through a right flank approach under local anesthesia [4], [5]. The CL was removed from each ovary, weighed and < 5 mm^3^ sections were snap-frozen in liquid N_2_ for subsequent protein and RNA analysis. Plasma progesterone concentrations were determined using the ImmuChem Progesterone DA Coated Tube radioimmunoassay kit (MP Biomedicals, Santa Ana, CA) with an intra-assay coefficient of variation of 9.13% and inter-assay coefficient of variation of 7.99%. The University of Nebraska-Lincoln Institutional Animal Care and Use Committee approved all procedures and facilities used in this animal experiment and animal procedures were performed in June 2009 (Control, 0.5, and 1 h) or October 2010 (2 and 4 h) at the University of Nebraska—Lincoln, Animal Sciences Department. Statistical differences in animal characteristics were determined using Kruskal-Wallis test followed by Dunn's post-test or one-way ANOVA followed by Bonferroni's multiple comparison test as appropriate (GraphPad Prism, La Jolla, CA).

### 2.2 Affymetrix bovine gene chip microarray

Luteal tissue from saline-treated (n = 3), and PGF2α treated animals [0.5 h (n = 3), 1 h (n = 3), 2 h (n=3), and 4 h (n = 3)] were homogenized and RNA was extracted using a Stratagene RNA Isolation Kit (Santa Clara, CA) following manufacturer's instructions. Transcriptional changes were analyzed by hybridization of 500 ng biotinylated cDNA using Affymetrix (Santa Clara, CA) bovine whole-transcript microarray (Bovine Gene v1 Array [BovGene-1_0-v1]; GPL17645) at the University of Nebraska Medical Center Microarray Core Facility. Comprehensive microarray methods and data was deposited in GEO database under accession GSE94069.

### 2.3 Microarray statistics

The microarray data were preprocessed using the robust multi-array average (RMA) method from Affymetrix expression console software (Affymetrix Inc., Santa Clara, CA) to normalize data at the exon level. The mean intensities of multiple probe sets of the same gene were calculated under each array to obtain the corresponding gene expression intensities. The data was filtered to keep the genes with a raw expression value after preprocessing to be 10 or more for at least three samples. Linear Models for Microarray Analysis (LIMMA) [6] in the Bioconductor suite [7] under the statistical program R [8] was applied to compare the log ratio between each of the PGF2α time points and the saline control after adjusting for the box effect. LIMMA applies a linear model and empirical Bayes method for assessing differential expression of the microarray data. Transcripts with a fold-change of at least 1.5 and a Benjamini-Hochberg adjusted *P*-value of less than 0.05 for each treatment condition versus control were identified as differentially expressed genes.

### 2.4 Self-organizing maps and statistics

Microarray data was filtered to keep genes with a raw expression value after preprocessing to be 30 or more for at least three samples. The log ratio between each of the time points and the saline control were compared using Linear Models of Microarray Analysis in the Bioconductor suite in R. The self-organizing map (SOM) clustering algorithm GeneCluster 2.0 [9] was applied to differentially expressed genes that had a greater than 1.5-fold change in expression and *P*-value ≤ 0.05 between PGF2α-treated samples and the saline control. The mean normalized log_2_ intensity values from each of the five examined biological conditions were used as transcript expression profiles in the clustering analysis. The number of iterations in SOM clustering was set to 500,000 to generate SOMs and hierarchical clustering (correlation-based distance, average link).

### 2.5 Dataset comparisons

Two previously published microarray datasets GSE23348 [2] and GSE27961 [3] examined the effect of *in vivo* PGF2α or analog treatment on the bovine luteal transcriptome using Affymetrix Bovine Whole Genome Gene Chips (GPL 2112). The datasets were chosen for comparison to the transcriptome dataset presented herein based on the use of a similar bovine gene array platform and similarities in the experimental protocol comparing mid-cycle control CL expression profiles to CL profiles after treatment with PGF2α analog for 4 h (GSE23348) or 6 h (GSE27961). Original.CEL and.CHP files were downloaded from the GEO database and processed as described above in the *Statistical Methods*. The differentially expressed mRNAs at 4 or 6 h were compared between the three microarray datasets to determine the similarities among the datasets.

### 2.6 Pathway analysis

Pathway analysis was evaluated using Ingenuity Pathway Analysis (IPA) [Application: Build: 430520M Copyright 2017 QIAGEN (Redwood City, CA)]. Transcripts found to be differentially expressed compared to saline-injected controls with > 1.5-fold change and *P* < 0.05 were input into IPA, DAVID, Panther, or STRING for bioinformatics analysis using Entrez gene IDs. Differentially expressed transcripts were analyzed in IPA using core analysis followed by comparison analysis between time points, or datasets. Unmapped genes in IPA were as follows: 0.5 h (20.6%), 1 h (8.7%), 2 h (11.7%), 4 h (13.3%), GSE94069 (12.6%, [1]), GSE23348 (9.8%, [2]), GSE27961 (8.0%, [3]) and common genes (6.5%). Data sets were assessed for prediction of upstream regulators and signaling pathways. Additional pathway analysis was completed using DAVID (Version 6.8, released: Oct 2016) [10], [11]; unmapped genes in DAVID were as follows: 0.5 h (0%), 1 h (1%), 2 h (1.7%), 4 h (1.2%), GSE94069 (0.7%, [1]), GSE23348 (0.8%, [2]), GSE27961 (0.7%, [3]) and common genes (no unmapped genes). The Panther database was used for gene annotations and comparison to other bioinformatics tools (Version 11.1, released: Oct 2016) [12], [13], [14]; unmapped genes in Panther were as follows: 0.5 h (34.5%), 1 h (28.2%), 2 h (35.5%), 4 h (38.9%), GSE94069 (39.5%, [1]), GSE23348 (31.6%, [2]), GSE27961 (29%, [3]) and common genes (24.2%). Finally, the STRING Database (Version 10.0, released: Apr 16, 2016) [15] was used to validate IPA findings and provide unique perspectives based on each tool's functionality.

Description of the methods are derived from the companion article [1] in Molecular and Cellular Endocrinology.

## Funding

This work was supported by the Agriculture and Food Research Initiative from the USDA National Institute of Food and Agriculture (NIFA) [2014–67011-22280 Pre-doctoral award to HT, 2011–67015-20076 to JSD and ASC, and 2013–67015-20965 to ASC, JRW and JSD]; USDA Hatch grants [NEB26-202/W2112 to ASC, eNEB ANHL 26–213 to ASC and JRW, NEB 26–206 to ASC and JRW]; USDA Agricultural Research Service Project Plan [3040–31000-093-00D to RAC]; the VA Nebraska-Western Iowa Health Care System Department of Veterans Affairs, Office of Research and Development Biomedical Laboratory Research and Development funds [BX000512 to JSD]; and The Olson Center for Women's Health, Department of Obstetrics and Gynecology, Nebraska Medical Center, Omaha, NE [JSD]; National Institute for General Medical Science (NIGMS) [INBRE - P20GM103427-14, COBRE - 1P30GM110768-01 to University of Nebraska Microarray Core and the Bioinformatics and Systems Biology Core]; and The Fred & Pamela Buffett Cancer Center Support [P30CA036727 to University of Nebraska Microarray Core and the Bioinformatics and Systems Biology Core].
