# Supplementary material for: Transcriptomic and bioinformatics analysis of the early time-course of the response to prostaglandin F2 alpha in the bovine corpus luteum
Source: Data Brief. 2017 Sep 1;14:695–706. doi: 10.1016/j.dib.2017.08.026 (PMC5596332; doi:10.1016/j.dib.2017.08.026)
Supplement: Supplementary file 2 — Supplementary material [file mmc1.pdf]

Conflicts of Interest Statement

Manuscript title:

Transcriptomic and Bioinformatics Analysis of the Early Time-course of the Response to Prostaglandin F2 alpha in the Bovine Corpus Luteum

The authors whose names are listed immediately below certify that they have NO affiliations with or involvement in any organization or entity with any financial interest (such as honoraria; educational grants; participation in speakers' bureaus; membership, employment, consultancies, stock ownership, or other equity interest; and expert testimony or patent-licensing arrangements), or non-financial interest (such as personal or professional relationships, affiliations, knowledge or beliefs) in the subject matter or materials discussed in this manuscript.

Author names:

Heather Talbott
Xiaoying Hou
Fang Qiu
Pan Zhang
Chittibabu Guda
Fang Yu
Robert A. Cushman
Jennifer R. Wood
Cheng Wang
Andrea S. Cupp
John S. Davis

The authors whose names are listed immediately below report the following details of affiliation or involvement in an organization or entity with a financial or non-financial interest in the subject matter or materials discussed in this manuscript. Please specify the nature of the conflict on a separate sheet of paper if the space below is inadequate.

Author names:

This statement is signed by all the authors to indicate agreement that the above information is true and correct (*a photocopy of this form may be used if there are more than 10 authors*):

Author's name (typed)	Author's signature	Date
Heather Talbott	Heather Talbott	July 3, 2017
Xiaoying Hou	[illegible]	7/5/17
Fang Qiu		
Pan Zhang	Pan Zhang	7/5/17
Chittibabu Guda		
Fang Yu		
Robert A. Cushman		
Jennifer R. Wood		
Cheng Wang		
Andrea S. Cupp		

This statement is signed by all the authors to indicate agreement that the above information is true and correct (*a photocopy of this form may be used if there are more than 10 authors*):

Author's name (typed)	Author's signature	Date
Heather Talbott		
Xiaoying Hou		
Fang Qiu	[illegible]	7/15/17
Pan Zhang		
Chittibabu Guda		
Fang Yu		
Robert A. Cushman		
Jennifer R. Wood		
Cheng Wang		
Andrea S. Cupp		

This statement is signed by all the authors to indicate agreement that the above information is true and correct (*a photocopy of this form may be used if there are more than 10 authors*):

Author's name (typed)	Author's signature	Date
Heather Talbott		
Xiaoying Hou		
Fang Qiu		
Pan Zhang		
Chittibabu Guda	G. [illegible]	7/3/17
Fang Yu		
Robert A. Cushman		
Jennifer R. Wood		
Cheng Wang		
Andrea S. Cupp		

This statement is signed by all the authors to indicate agreement that the above information is true and correct (*a photocopy of this form may be used if there are more than 10 authors*):

Author's name (typed)	Author's signature	Date
Heather Talbott		
Xiaoying Hou		
Fang Qiu		
Pan Zhang		
Chittibabu Guda		
Fang Yu	Yu [signature]	7/3/17
Robert A. Cushman		
Jennifer R. Wood		
Cheng Wang		
Andrea S. Cupp		

This statement is signed by all the authors to indicate agreement that the above information is true and correct (*a photocopy of this form may be used if there are more than 10 authors*):

Author's name (typed)	Author's signature	Date
Heather Talbott		
Xiaoying Hou		
Fang Qiu		
Pan Zhang		
Chittibabu Guda		
Fang Yu		
Robert A. Cushman	[signature]	JULY 11, 2017
Jennifer R. Wood		
Cheng Wang		
Andrea S. Cupp		

This statement is signed by all the authors to indicate agreement that the above information is true and correct (*a photocopy of this form may be used if there are more than 10 authors*):

Author's name (typed)	Author's signature	Date
Heather Talbott		
Xiaoying Hou		
Fang Qiu		
Pan Zhang		
Chittibabu Guda		
Fang Yu		
Robert A. Cushman		
Jennifer R. Wood	Jennifer R Wood	7/5/17
Cheng Wang		
Andrea S. Cupp	Andrea Cupp	7/5/17

This statement is signed by all the authors to indicate agreement that the above information is true and correct (*a photocopy of this form may be used if there are more than 10 authors*):

Author's name (typed)	Author's signature	Date
Heather Talbott		
Xiaoying Hou		
Fang Qiu		
Pan Zhang		
Chittibabu Guda		
Fang Yu		
Robert A. Cushman		
Jennifer R. Wood		
Cheng Wang	Cheng Wang	07-06-2017
Andrea S. Cupp		

This statement is signed by all the authors to indicate agreement that the above information is true and correct (*a photocopy of this form may be used if there are more than 10 authors*):

Author's name (typed)	Author's signature	Date
John S. Davis		
